# Supplementary material for: Assessment tools for attention deficits in patients with stroke: a scoping review across components and recovery phases
Source: PeerJ. 2025 Mar 27;13:e19163. doi: 10.7717/peerj.19163 (PMC11955193; doi:10.7717/peerj.19163)
Supplement: Supplemental Information 2 [file peerj-13-19163-s002.docx]

**Supplementary file 1**

Search terms;

**PubMed**

(“Stroke”[MeSH Terms] OR “Stroke”[Title/Abstract] OR “Hemiplegia”[MeSH Terms] OR “Hemiparesis”[MeSH Terms]) AND (“Attention disorder”[MeSH Terms] OR “Attention deficits”[MeSH] OR “Attention deficits”[Title/Abstract]) AND “Rehabilitation”[MeSH]

**Web of Science**

“(Stroke OR Hemiplegia OR Hemiparesis) AND (Attention disorder OR Attention deficits) AND Rehabilitation”

**CINAHL**

“(Stroke OR Hemiplegia OR Hemiparesis) AND (Attention disorder OR Attention deficits) AND Rehabilitation”

**Ovid**

“(Stroke OR Hemiplegia OR Hemiparesis) AND (Attention disorder OR Attention deficits) AND Rehabilitation”
